# Phytochemical Profile and Antioxidant Properties of Bee-Collected Artichoke (*Cynara scolymus*) Pollen

**DOI:** 10.3390/antiox10071091

**Published:** 2021-07-07

**Authors:** Aleksandar Ž. Kostić, Danijel D. Milinčić, Nebojša Nedić, Uroš M. Gašić, Bojana Špirović Trifunović, Denis Vojt, Živoslav Lj. Tešić, Mirjana B. Pešić

**Affiliations:** 1 Faculty of Agriculture, University of Belgrade, Nemanjina 6, 11080 Belgrade, Serbia; danijel.milincic@agrif.bg.ac.rs (D.D.M.); nedicn@agrif.bg.ac.rs (N.N.); spirovic@agrif.bg.ac.rs (B.Š.-T.); deniswojt@gmail.com (D.V.); mpesic@agrif.bg.ac.rs (M.B.P.); 2 Institute for Biological Research “Siniša Stanković”, National Institute of Republic of Serbia, University of Belgrade, Bulevar Despota Stefana 142, 11060 Belgrade, Serbia; uros.gasic@ibiss.bg.ac.rs; 3 Faculty of Chemistry, University of Belgrade, Studentski Trg 12-16, 11000 Belgrade, Serbia; ztesic@chem.bg.ac.rs

**Keywords:** bee-collected pollen, artichoke, phenolics, fatty acids, carotenoids, antioxidant properties

## Abstract

The current study intended to determine, for the first time, phenolic and fatty acid profile, antioxidant and certain nutritional properties of monofloral bee-collected artichoke (*Cynara scolymus*) pollen. Based on UHPLC-DAD MS-MS analysis the main phenolics in extractable fraction were different flavonol glycosides (in particular Isorhamnetin-3-*O*-glucoside, 49.2 mg/kg of dry weight) while ferulic acid was the predominant phenolic compound (39.4 mg/kg of dry weight) in the alkaline hydrolyzable fraction. Among fatty acids (FAs), results of GC-FID analysis revealed prevalence of unsaturated FAs with *cis*-5,8,11,14,17-eicosapentaenoic acid (EPA) and oleic acid as the main ones- 28.4% and 24.9%, respectively. Based on the FA composition, nutritional analysis proved that artichoke bee-collected pollen had balanced ω-6 and ω-3 FAs content. To determine the antioxidant properties of pollen, five different assays were applied. It was proved that bioactive compounds in artichoke pollen possessed significant ability to quench DPPH radical as well as ABTS radical cation. In addition, in vitro phosphomolybdenum assay confirmed that artichoke pollen is an excellent source of different antioxidants. Pollen extracts exhibited moderate ferric reducing power as well as low ferrous chelating ability. Some further antioxidant studies (preferably in vivo) should be performed to confirm the observed results.

## 1. Introduction

Bioactive compounds (phenolics, carotenoids, unsaturated fatty acids, etc.) are considered as health promoting agents due to their potential positive effect on human health. In order to ensure regular intake of bioactives and to improve quality of some ordinary food, there are different functional ingredients that can be prepared/used. One of the most promising is pollen. This plant product can be obtained as floral (collected by hands from flower) or as bee-collected which is the form commercially available on the market. Pollen has been recognized as an excellent functional food and feed ingredient [1] as well as a good source of different bioactive compounds [2,3]. Chemical composition of pollen is strongly influenced by its botanical [4,5,6,7,8] and geographical origin [9]. In case of bee-collected pollen it is also important to obtain a monofloral sample (more than 80% of one pollen grain type) [10] in order to ensure consistent and stable chemical contexture. However, sometimes it is quite hard to prepare and/or collect the monofloral sample since bees rarely visit only one plant species during pasture. Phenolics are one of the most important secondary plant metabolites which are regularly present in pollen. In addition to the chemical structure, they can be differentiated by the fact that they are present as free (extractable with different solvents) or hydrolyzable forms in plant cells. According to literature, extractable phenolics are predominantly present in the vacuoles while hydrolyzable phenolics (insoluble fraction during extraction with different solvents) are mostly localized in cell wall matrices [11]. Considering their diversity, it is observed that glycosides of flavonoids and phenolic acids are the predominant type of phenolic compounds registered in pollen. It was reported that these sugar-phenolic compound complexes can make even more than 65% of total phenolics in sunflower bee-collected pollen [7]. All dietary important plant flavonoids can be found either as *O*-glycosides or *C*-glycosides [12]. Possible reasons for forming these complex structures are having higher solubility in water and having a higher melting point which allows biosynthesis of different precursors of volatile compounds [13]. In addition, by forming flavonoid complexes with other compounds plants can enable possible detoxification of the organism [13]. Furthermore, it is possible that some flavonoid glycosides such as naringin can act as repellents of predators since they are the main source of bitter taste of several citruses such as grapefruit and orange [14]. According to literature, the most abundant glycoside in pollen is rutin [15] which can be used as a parameter of pollen freshness together with the content of proline [16,17]. Furthermore, it has been proven that phenolics are useful chemotaxonomic tools to make a connection between plant and botanical origin of pollen [18,19]. Similarly, some fatty acids (FAs) can also be used as a marker of botanical origin of pollen [20]. Moreover, fatty acids are an important parameter for determination of nutritional properties of food since they can be separated in two important subclasses- saturated fatty acids (SFA) and unsaturated fatty acids (UFA) as well as ω-6 and ω-3 FAs belonging to UFA. For some monofloral bee-collected pollen samples, precise phytochemical profiles have been reported such as in the case of Indian mustard (*Brassica juncea*) [4], sunflower (*Helianthus annuus*) [7,21], *Alternanthera*, *Cocos nucifera*, *Myrcia*, *Mimosa* sp., *Anadenanthera* [6], *Rhododendron ponticum* [22] bee-collected pollen, etc. In addition to extractable fraction, which is easily available via simple extraction performed with different solvents, a part of plant phenolics (in some cases it can be a significant part) is present in the form of so-called nonextractable phenolics which can be divided in two fractions: hydrolyzable phenolics and nonextractable proanthocyanidins [23]. However, there is still a lack of data about hydrolyzable fraction of phenolics in pollen since most of the reports actually monitored just extractable phenolic fractions. To the best of our knowledge, the first report about hydrolyzable phenolic fraction (authors referred to it as insoluble-bound) was recently made for raw pine pollen from China [24] as well as for commercially obtained pollen samples from Turkey [25]. Authors pointed out that the hydrolyzable fraction was predominant compared to the extractable fraction. In addition, for pollen produced from some bee pastures, there is still insufficient data about the phytochemical profiles. One of the most important properties of pollen is good antioxidant activity [3,7,21,25,26,27,28] which can be connected to its suitable chemical composition and above all, the presence of different phenolic compounds. According to literature, the main antioxidants in pollen are small molecular weight compounds mostly phenolics and vitamin C as hydrophilic components, and vitamin E as lipophilic antioxidants [15]

Artichoke (*Cynara scolymus*) is a cultivated variety of the wild cardoon (*C. cardunculus*) firstly grown and applied in the Mediterranean region. It is a perennial plant with favored anemochory dissemination, blooms in July enriching diversity of melliferous plants in that period. However, recently it has been determined that the reproduction process of artichokes is significantly improved by involving entomophilous pollination via honeybees [29].

Based on all available data, the aims of this research were:

(a) to determine, for the first time, general (total extractable and alkaline hydrolyzable phenolics, total extractable and alkaline hydrolyzable flavonoids, total extractable and alkaline hydrolyzable dihydroxycinnamic derivatives, total carotenoids) as well as a detailed phytochemical profile (extractable and alkaline hydrolyzable phenolics and fatty acids) of bee-collected artichoke pollen.
(b) to determine its antioxidant properties based on five different assays- total antioxidant capacity (TAC), ferric reducing power (FRP), ABTS radical cation and DPPH radical scavenging activities as well as Fe^2+^ chelating capacity (FCC).
(c) to determine two nutritional parameters (UFA/SFA ratio, ω-6/ω-3 ratio) based on the presence of saturated/unsaturated fatty acids.

## 2. Materials and Methods

### 2.1. Collection of Bee-Collected Artichoke Pollen

Pollen sample was collected during the blooming period of the artichoke flower-heads on the Experimental farm of Radmilovac of the Faculty of Agriculture, University of Belgrade (44°75′41.38″ and 20°58′12.44″) in July 2020. A full bee colony in ten-frames Langstrot Rut hive with a year-old queen bee was chosen on an apiary and a pollen trap collector was installed at the entrance of the beehive. After at least 10% of artichoke flower-heads had bloomed, the bee colony was placed under an isolation cage in order to prevent bees to visit any other plants during pollen collection. Following the activation of the pollen trap collector, pollen was collected every day and the collectors were thoroughly cleaned. Pending the analysis, pollen was kept in a freezer in plastic vacuum bags wrapped in aluminum at the temperature of −20 °C. The sample for analysis was prepared from pellets, obtained from pollen trap, with the same color. A representative sample was prepared by careful mixing of collected pollen for equalization.

### 2.2. Preparation of Pollen Extracts

**Figure 1:**
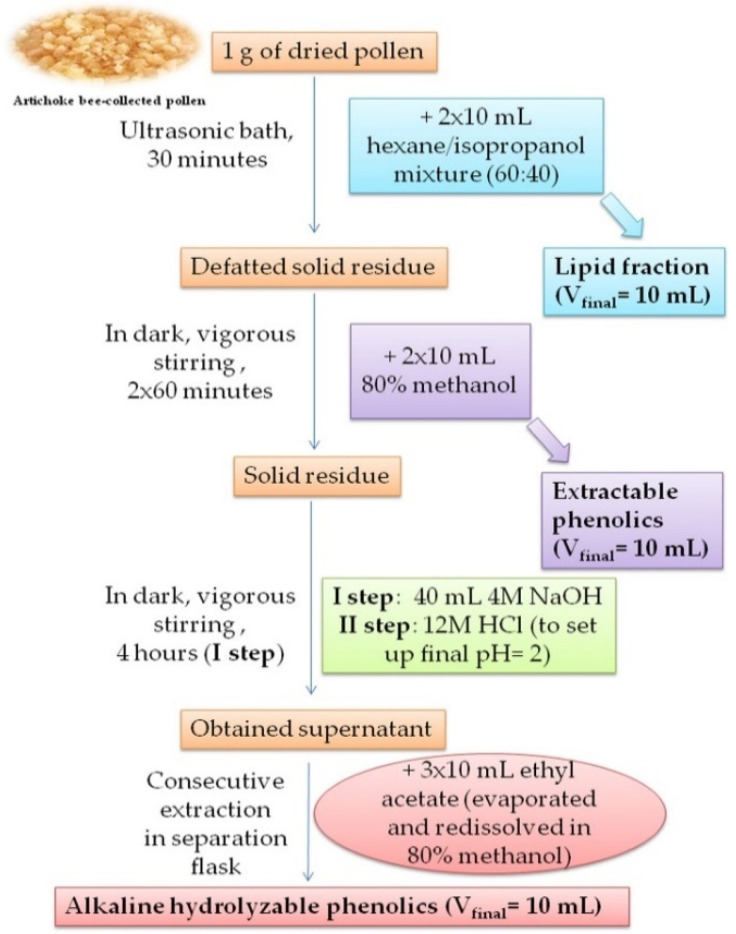
The sheme of extraction procedure.

A scheme of extraction procedure is summarized in Figure 1 and explained in detail in further text. For the preparation of lipid fraction, bee-collected pollen (1 g) was extracted with 10 mL of hexane/isopropanol (60:40) mixture as it was suggested in literature [30].

Extraction was performed for 30 min in an ultrasonic bath (VAB SB 3 LD, maximum power 440 W, operating frequency 40 Hz). After centrifugation, the supernatant was separated and the extraction procedure was repeated with another 10 mL of solvent mixture with vigorous stirring. The resulting supernatant was combined with the previous one, the solvents were evaporated to dryness at room temperature under a nitrogen atmosphere and the residual lipid fraction was finally dissolved in 10 mL of the mixture. By doing so, a lipid extract of bee-collected artichoke pollen was obtained, and it was ready for further GC-FID analysis. The extraction of lipids was performed separately for GC/FID analysis and for the determination of antioxidant properties. Hexane/isopropanol was used for extraction in both procedures. However, the preparation of the lipid fraction to determine the antioxidant properties additionally included rotary evaporation to dryness and final reconstitution in 10 mL of ethanol [31]. Defatted solid residue, which remained after the lipid extraction, was used for further extraction of phenolic compounds. For that purpose, 10 mL of 80% methanol (MeOH) was added and the extraction of free (extractable) phenolic fraction was performed for 60 min with vigorous stirring in a light-protected cuvette. After centrifugation, the supernatant was then separated and the extraction procedure was repeated. The two supernatants were combined, the solvent was evaporated to dryness and the residual solid was redissolved in 10 mL of 80% MeOH. Thus, an extractable phenolic fraction was obtained. This extract was used for further testing of this fraction. The solid residue remained after extractable phenolic fraction separation was used for preparation of alkaline hydrolyzable phenolic extract. Meaning, alkaline digestion was performed [32] by using 40 mL of 4 M NaOH solution and the extraction was performed for 4 h. After the extraction was completed, the obtained extract was neutralized with the required volume of 12M HCl until the pH of the solution was 2. Thereafter, the released phenolics were extracted three times with 10 mL of ethyl acetate. All supernatants were combined, the solvent was evaporated to dryness and the residual fraction was finally dissolved in 10 mL of 80% MeOH. In this way, an extract of alkaline hydrolyzable phenolics was prepared, which was used for further analyses.

### 2.3. Determination of Total Phenolic, Flavonoid and Dihydroxycinnamic Derivative Content

The total phenolic (TPC) and flavonoid (TFC) content of extractable and alkaline hydrolyzable methanolic extract of bee-collected artichoke pollen was determined using well known colorimetric assays, as previously described [7]. For TPC determination properly diluted extracts (140 μL) were mixed with 600 μL of Folin-Ciocalteu working solution and put in a dark place. After 5 min, 460 μL of sodium carbonate solution (7.5%) was added. Mixture was left in the dark for 90 min to develop color. After that period the absorbance of made samples was measured at 765 nm against the prepared blank which contained milliQ water instead of sample aliquot. In order to assess TFC values, non-diluted extracts (125 μL) were mixed with 625 μL of distilled water and 37.5 μL of 5% sodium nitrite solution. After 6 min, 75 μL of 10% aluminum chloride solution was mixed and the samples were left in the dark for additional 5 min to react. After that period 250 μL of sodium hydroxide solution and 138 μL of distilled water were added and the mixtures were shaken. The intensity of developed color was measured at 510 nm, against the prepared blank which contained milliQ water instead of sample aliquot. The TPC and TFC of the sample were expressed as gallic acid (mg GAE/g) and quercetin (mg QE/g) equivalents per g of dry weight of pollen powder (in further text dry weight), using previously prepared calibration curves.

Dihydroxycinnamic derivatives in the extractable and alkaline hydrolyzable fractions of pollen were estimated as previously described [33] with some modifications. Briefly, 0.2 mL of aliquot was mixed with 0.4 mL of 0.5 M HCl, 0.4 mL of Arnow’s reagent, 0.4 mL of 2.125M NaOH and 0.6 mL milliQ water, respectively. After incubation for 20 min [34] at room temperature in the dark, absorbance of mixture was measured at 525 nm. Results were expressed as chlorogenic acid equivalents per g of dry weight (mg CGAE/g DW).

### 2.4. Determination of Total Carotenoid Content

Total carotenoid content in the pollen sample was determined using the previously described method [35]. About 0.5 g of the earlier ground pollen was extracted with 5 mL of 80% acetone, with constant stirring in a shaker for 12 h in the dark. Afterwards, 5 mL of extraction agent was added to the mixture and the sample was further extracted for another 12 h. Upon extraction, the sample was centrifuged, and the supernatant was collected. Absorbance was measured directly from the prepared supernatant at 450 nm. The total carotenoid content was determined using the following equation [36]:

µg carotenoid/g DW sample = (A∙V∙10^6^)/(E_1cm_∙100∙m) (1)

where A represents the absorbance of the supernatant, V is the total volume of the extract (10 mL), E_1cm_ is the extinction coefficient for the used solvent (2500) and m is the measured mass of the sample.

### 2.5. UHPLC-DAD MS/MS Analysis of Pollen Fractions

The detailed profile of phenolic compounds in both prepared fractions of bee-collected artichoke pollen was determined via UHPLC-DAD MS/MS analytical technique as it is described in our previous researches [37,38]. Namely, the Dionex Ultimate 3000 UHPLC system with a diode array detector (DAD) and TSQ Quantum Access Max triple-quadrupole (QqQ) mass spectrometer (ThermoFisher Scientific, Basel, Switzerland) was used for the phenolic compounds quantification. The elution was performed at 40 °C on a Syncronis C18 column (100 × 2.1 mm, 1.7 μm particle size) with mobile phase consisted of (A) water +0.1% formic acid (*v*/*v*), and (B) acetonitrile (MS grade) +0.1% formic acid (*v*/*v*), which were applied in the gradient elution [37]. The parameters of QqQ mass spectrometer equipped with an heated electrospray ionization (HESI) source was previously described [37]. The time-selected reaction monitoring (tSRM) experiments for quantitative analysis were performed using two MS^2^ fragments for each compound that were previously defined as dominant in the product ion scanning (PIS) experiments. The phenolics were identified via direct comparison with commercial standards.

### 2.6. GC-FID Analysis of Fatty Acid Content in Pollen with Nutritional Assessment

Analysis of fatty acids was performed in hexane-isopropyl alcohol extract (described in Section 2.3) via GC-FID analysis. Briefly, the obtained lipid extract was evaporated under nitrogen flow and redissolved in 1 mL of hexane. Afterwards, conversion of fatty acids in fatty acid methyl esters (FAME’s) was performed by using 1 mL of 14% boron trifluoride/methanol reagent. The obtained FAMEs were determined by using capillary gas chromatography (GC) analysis on Agilent Technologies 6890 apparatus equipped with flame ionization detector (FID). Details about analytical procedure and applied conditions are presented in previous work [39]. In order to determine nutritional potential of pollen based on different fatty acid content the following nutritional parameters were calculated according to the given equations:

UFA/SFA ratio = (total content of saturated FAs)/(total content of unsaturated FAs)(2)

ω-6/ω-3 ratio = (total content of ω-6 FAs)/(total content of ω-3 FAs)(3)

### 2.7. Antioxidant Properties of Bee-Collected Artichoke Pollen

For examination of antioxidant properties lipid, extractable phenolic and alkaline hydrolyzable phenolic fractions were used. Determination of ABTS radical cation scavenging activity, total antioxidant capacity and ferrous-ion-chelating capacity were analyzed according to previously described methods [38]. For ABTS radical cation assay, 100 mL of lipid, extractable and alkaline hydrolyzable phenolic extracts of bee-collected artichoke pollen was mixed with 1 mL of ABTS**^∙+^** working solutions whose absorbance was in the range of 0.7–0.8. After 7 min of vigorous stirring, absorbance was measured at 734 nm. Obtained results were used to calculate the percentage of quenched radicals according to the following equation:

ABTS radical cation scavenging activity (%) = (Ac − As)/Ac × 100(4)

where Ac is the absorbance of ABTS radical cation working solution, As is the absorbance of the sample mixed with ABTS radical cation working solution.

For the determination of TAC 0.3 mL of samples was mixed and incubated (90 °C, 90 min) with 3 mL of phosphomolybdenum reagent. After that, the samples were cooled, and the absorbance was measured at 695 nm. The obtained results of pollen fractions were expressed as mg of ascorbic acid equivalents per g of dry weight sample (mg AAE/g DW).

For FCC, 200 mL of pre-prepared pollen fractions was mixed with milliQ water and 2 mM iron(II) sulfate. After 30 min, 5 mL of ferrozine was added and the absorbance of the mixture was measured after 10 min at 562 nm. Results for Fe^2+^ chelating capacity (%) were calculated according to the following equation:

FCC = ((Ac − As)/Ac) × 100(5)

where Ac is the absorbance of the blank; As is the absorbance of samples.

In addition to ABTS radical cation scavenging activity of pollen extracts DPPH radical assay was also applied. For that purpose, 105 μL of original extracts was mixed with 840 μL of DPPH**^∙^** working solution (0.062 g/100 mL) prepared in methanol according to literature data [40]. Samples were left in the dark for 30 min to react with DPPH radical at room temperature. After that, the resulting discoloration of DPPH radical working solution was determined by reading the absorbance at 517 nm. The obtained results were expressed as % of inhibition of DPPH radical and calculated according to the following equation:

DPPH radical scavenging activity (%) = (Ac − As)/Ac × 100(6)

where Ac is the absorbance of DPPH radical working solution, As is the absorbance of sample mixed with DPPH radical working solution.

In order to determine the ability of extracts to participate in redox processes, FRP was determined according to the previously described method [41] with some modifications. Briefly, 0.5 mL of original extracts was mixed with 0.5 mL of phosphate buffer solution (pH = 6.6) and 0.5 mL of 1% solution of K_3_[Fe(CN)_6_]. After heating for 20 min (t = 50 °C) 0.5 mL of 10% trichloroacetic acid solution was added to samples. After centrifugation 0.5 mL of clear supernatant was mixed with the same volume of distilled water and 0.1 mL of 0.1% ferric-chloride solution. The obtained green color of solution was read at 700 nm. The results were expressed as mg of AAE/g of DW.

### 2.8. Statistical Analysis

Statistical analysis was conducted using the Statistica software version 12.0 (StatSoft Co., Tulsa, OK, USA). All experiments were performed in triplicate and results expressed as means ± standard deviation (SD). To test the significance of differences between means, *t*-test was applied at *p* < 0.05. Figures were drawn in GraphPad Prism6 software (San Diego, CA, USA).

## 3. Results and Discussion

In order to determine a detailed phytochemical profile in bee-collected artichoke pollen several parameters were monitored and ascertained.

### 3.1. Total Phenolic, Flavonoid, Dihydroxycinnamic Derivative and Carotenoid Content

The content of total phenolics, flavonoids, dihydroxycinnamic derivatives and carotenoids was determined and presented in Table 1.

As it can be observed from Table 1 the most dominant fraction among extractable compounds in pollen were phenolics (5.3 mg/g GAE DW) followed by dihydroxycinnamic acid derivatives (1.1 mg/g CGAE DW) and flavonoids (0.81 mg/g QE DW). On the other hand, alkaline hydrolyzable fraction was significantly reduced compared to extractable fraction and it contained predominantly flavonoids (0.96 mg/g QE DW) followed by phenolics (0.50 mg/g GAE DW) while the dihydroxycinnamic acid derivatives in this fraction were absent. The obtained results for extractable TPC and TFC were in line with [7] or higher [21] than the previously published data for monofloral sunflower bee-collected pollen from Serbia and Slovakia, respectively. To the best of our knowledge, there are no data for determination of dihydroxycinnamic acid derivatives in bee-collected pollen. The observed absence of dihydroxycinnamic acid derivatives in alkaline hydrolyzable fraction via spectrophotometric determination is also in line with results obtained through HPLC analysis presented later. Based on the results for total carotenoids (5.0 μg/g DW) artichoke pollen cannot be considered as a good source of these compounds compared to some other monofloral pollen samples such as sunflower bee-collected pollen from Slovakia [21] or samples collected in Romania with different botanical origin [42]. However, the observed low content of carotenoids is in line with visual appearance of the collected pollen since it was almost white with some pale yellowish color (Figure 1).

### 3.2. The Phenolic Profile of Bee-Collected Artichoke Pollen

In order to obtain more detailed and reliable data for the content of different phenolics present in the pollen sample, HPLC DAD MS/MS analysis was performed. The results are given in Table 2 while UV chromatograms at 280 nm are given as a supplementary file (Figure S1). However, only MS data (Table S1) were used for both identification and quantification of phenolic compounds.

Among monitored phenolic compounds (available through appropriate standards) 10 different phenolic compounds were confirmed and quantified. The most predominant compound in the extractable fraction was Isorhamnetin 3-*O*-glucoside. Furthermore, three additional flavonoid glycosides (Quercetin-3-*O*-glucoside, Quercetin-3-*O*-rutinoside, Kaempferol-3-*O*-glucoside) and one aglycone (Kaempferol) were quantified making more than 90% of all quantified phenolics in the extractable fraction. In addition, extractable phenolic fraction was predominant comprising more than 60% of total phenolics in pollen sample. The obtained results are in line with literature confirming that pollen contained predominantly flavonoids in the form of glycosides followed by aglycone form [7,21,43]. Regarding the presence of Isorhamnetin 3-*O*-glucoside Mihajlović et al. [44] detected the same compound in floral pollen from *Ambrosia artemisiifolia* L. collected in Serbia. This glycoside was the second most dominant phenolic acid in bee-collected monofloral pollen originated from *Mimosa caesalpiniaefolia* (Brazil) [6]. Similar Isorhamnetin derivatives (Isorhamnetin-di-3,7-*O*-glucoside, Isorhamnetin-3-*O*-(2”,3”-*O*-dirhamnosyl)-glucoside, Isorhamnetin-3-*O*-(2″-*O*-rhamnosyl)-glucoside) were detected in bee-collected monofloral pollen originated from *Cocos nucifera* obtained from Southern Brazil [5] but without data about quantities. Caffeic acid was the only phenolic acid quantified in the extractable fraction. It was also found in monofloral bee-collected pollen obtained from *Zea mays* [27] but in significantly higher amount (4.2 mg/g) confirming the importance of botanical origin of pollen for its chemical composition. In general, caffeic acid is one of the most common phenolic acids found in bee-collected pollen alongside gallic, ferulic and cinnamic acids [15]. However, HPLC analysis of alkaline hydrolyzable phenolic fraction revealed quite interesting results. It was observed that the content of glycosides and aglycones was significantly reduced (more than 10 times) compared to the extractable fraction. On the other hand, the content of phenolic acids was significantly higher since ferulic acid was quantified as the most predominant compound in the alkaline hydrolyzable fraction although this compound was not detected in the free fraction. Caffeic acid, as the second phenolic acid was not detected in the alkaline hydrolyzable fraction which was in line with the results obtained from spectrophotometric determination given in Section 3.1. These intriguing results for alkaline hydrolyzable fraction can be connected with aggressive surroundings used during the extraction of alkaline hydrolyzable phenolics where strong alkaline and acidic conditions were applied. As a result, diminished content of glycosides is observed since they are sensitive and can be degraded under these conditions. The first product of this degradation process is aglycone [45,46] but it can also be further transformed in phenolic acids such as ferulic acid [46]. In addition, it is possible that this acid was bound to the plant cell wall, as it is often the case in different food matrices [11], and therefore was not detectable in the extractable fraction. In our previous research [7] the absence of ferulic acid in the methanolic extract of sunflower bee-collected pollen was also observed but this acid was detected in the ethanolic extract obtained from the same sample which can be related to different polarity of used solvents. The obtained results for detected flavonols in extractable fractions were in line with previously published research but with different amounts of quantified glycosides. Among the rest of quantified phenolics, Apigenin and its glycoside, Apigetrin were detected in measurable quantities- a summary about 4 mg/kg in the extractable fraction. Based on literature search, this is the first report about the presence of Apigetrin (Apigenin-7-*O*-glucoside) in bee-collected pollen. This flavone and its glucoside have been recognized as potential nutraceuticals as well as health promoting agents with a beneficial role in treating several diseases such as diabetes, amnesia, Alzheimer’s disease, etc. [47]. Comparing the data from the current study with available literature data for phytochemical analysis of different parts of artichoke (leaves, outer bracts, heads and stems) some similarities as well as differences can be observed. Namely, caffeic acid derivatives were observed as the main phenolic compounds followed by apigenin derivatives [48] which was similar to the given study. Namely, caffeic acid, ferulic acid and apigenin-7-*O*-glucoside were found in artichoke pollen. Authors also reported different luteolin derivatives detected in artichoke plant [48] which were not found in examined pollen samples. Furthermore, in this review article there are no data about the presence of quercetin and isorhamnetin derivatives which were found in the current study which can be perhaps recognized as a specificity of pollen as material. Moreover, it can be concluded that, in contrast with some other pollen samples, artichoke pollen was characterized neither by great diversity nor high quantity of phenolic compounds. However, it should not be overlooked that some of the compounds were not available for determination due to a lack of appropriate standards. Due to this, some further investigation based not just on quantitative but also on qualitative analysis should be performed. For instance, a detailed phenolic profile obtained with UHPLC-Orbitrap MS analysis will bridge a possible data gap.

### 3.3. The Fatty Acid Profile of Bee-Collected Artichoke Pollen with Nutritional Assessment

Lipids are an important part of pollen grain since it can be found in all fragments of grain including sporopollenin–the biopolymer located in the exine membrane–which contains different fatty acids (FAs) and carotenoids as lipophilic components [49].

In addition, fatty acids are part of neutral lipid fraction triacylglycerols (TAGs) present in lipid droplets as inner developed components [49]. In contrast with the lipid bilayer in cell membranes which consists almost exclusively of C16 and C18 FAs, great diversity of FAs can be found in storage and reproductive parts of plants. In order to characterize lipophilic part of pollen a detailed FA profile has been determined and presented in Table 3. It can be observed that artichoke pollen contained 11 different FAs among 37 monitored FAs. Two FAs stood out regarding the determined content: *cis*-5,8,11,14,17-eicosapentaenoic acid (C20:5n3), also known as EPA, was the most represented FA (28.4%) followed by oleic acid (C18:1n9c) which made 24.9% of total FAs. Whereas, palmitic acid (C16:0) was detected only in traces—0.5%. The great prevalence of unsaturated fatty acids (UFA) was observed (76.32%) compared to total saturated fatty acid (SFA) content which was 23.66%. Based on this, UFA/SFA ratio, as useful nutritional parameter, can be calculated. In this case it had the value of 3.23 which is quite above the recommended value of 1.6 [39]. The significant presence of EPA is quite interesting and important in this case. To the best of our knowledge, this FA is rare in previously published reports about bee-collected pollen. Actually, we managed to find only one report where authors determined occurrence of EPA in bee-collected pollen from India originated from coconut, coriander and rapeseed [50]. However, only coconut pollen contained EPA in significant quantity (33.6%) comparable with artichoke pollen. According to literature, the main sources of this fatty acid in human diet are algae and some water plants [51] while in some usual plant-based foods it is not found in a regular way. This makes artichoke pollen more interesting from the nutritional point of view. EPA is an important long-chain ω-3 FA with positive effect on our health especially for regular fetal growth and proper aging [51] while the most recently published review article emphasized its importance in the reduction of inflammation processes [52]. However, the same authors clearly stated that further experimental clinical trials are necessary in order to determine precise influence of EPA on health of some specific groups of patients [52] and potential consumers.

In some cases, there are FAs which can be used as potential chemotaxonomic markers for some plant species/genus/families. For instance, authors made a connection between occurrence of isopalmitic (14-methylpentadecanoic) acid in bee-collected pollen samples from Spain with the presence of pollen belonging to the plants from *Rubus* genus [53]. In addition, PCA analysis for fatty acid profile of Serbian bee-collected pollen revealed a potential connection between pollen originated from *Zea mays* with the presence of 2 unsaturated FAs—palmitoleic acid (C16:1) as well as eicosadienoic acid (C20:2), so-called rare FA [20]. Since EPA was not regularly found in bee-collected pollen some further and additional explorations are required in order to determine whether it can be considered as some useful tool for botanical discrimination of the origin of pollen.

Based on data for FA composition of bee-collected artichoke pollen it was possible to determine several useful nutritional parameters which are related to lipids as ambiguous components of food-desirable/useful and undesirable/non-useful. Alongside proteins and sugars, lipids are an irreplaceable part of diet. However, excessive intake of some lipids such as FAs, TAGs, sterols, etc. can lead to development of several diseases (especially some cardiovascular diseases and Type 2 diabetes) as well as obesity. In case of FAs, in contrast with suitable prevalence of UFA compared to SFA in our diet, the most recent data revealed necessity for balanced intake of ω-6 and ω-3 FAs with suggested ω-6/ω-3 ratio about 1–2 [54]. Currently, in western diets there is strong prevalence of ω-6 FAs. However, bee-collected artichoke pollen revealed that there is no dominance of ω-6 FAs but a slight prevalence of ω-3 FAs with ω-6/ω-3 ratio about 0.4. This is certainly a desirable value compared to some other food ingredients with significant prevalence of ω-6 FAs including some other pollen types. For instance, floral maize pollen obtained from different maize varieties from Serbia had ω-6/ω-3 ratio values between 4.2 and 23.6 [39]. In this case it is clear that the botanical origin of pollen played a crucial role.

### 3.4. Antioxidant Properties of Bee-Collected Artichoke Pollen

Knowing the antioxidant properties of food represent one of the usual analyses performed on different food sources in order to determine their potential health promoting possibilities. However, it is important to apply different antioxidant assays since none of them can give complete information. Thus, the current study used five different assays (ABTS radical cation and DPPH radical quenching, TAC, FRP and FCC) and the results are presented in Table 4.

According to the results for TAC (Table 4) significant differences were observed between extractable and alkaline hydrolyzable phenolic fraction extracts. Extractable fraction has proved to be an excellent source of antioxidants while alkaline hydrolyzable fraction had significantly lower capacity. There are rare reports for TAC values of bee-collected pollen in literature. High value for TAC can be related not only to monitored phenolics but also to the presence of vitamin C [55] which was not analyzed in the current study but can be present in pollen in significant quantities [26]. The obtained results for both fractions were significantly higher compared to bee-collected pollen (wild carrot, rosemary and eucalyptus monofloral samples and polyfloral samples) from Algeria where authors determined that TAC values were in the range from 0.072 to 0.102 mg/g GAE [56] while the result for extractable fraction was slightly higher compared to Moroccan monofloral bee-collected pollen samples- 3.9 to 9.7 mg/g AAE [57]. Lipid fraction of bee-collected pollen had significantly higher TAC value compared to alkaline hydrolyzable fraction but lower compared to extractable phenolics. It is possible that lipophilic antioxidants such as vitamins A and E (not quantified in research) together with carotenoids and unsaturated fatty acids made contribution to the observed result for lipid fraction.

Ferric reducing power of both phenolic fractions is related to the ability of pollen compounds to reduce Fe^3+^ ion via donation of electron. In addition to the phenolic compounds, reduction of ferric ion can be caused by reducing sugars [55]. In that sense, the observed increased activity of extractable phenolic fraction (468.6 mg/kg AAE) compared to alkaline hydrolyzable fraction (115.1 mg/kg AAE) is expected since the first one also contained reducing sugars extracted with 80% methanol. Contrary to the phenolic fractions, lipid fraction did not exhibit any ability to reduce ferric ions. The observed result for lipid fraction was in line with the result from literature where *Pluchea* leaves hexane extract possessed negligible FRP (0.02 mg/g GAE) [58].

ABTS radical cation and DPPH radical quenching abilities are the most frequently utilized antioxidant assays providing data about capability of analyzed food to prevent formation of free radicals under oxidative stress conditions. Based on the obtained results it can be observed that extractable and alkaline hydrolyzable phenolic extracts revealed quite interesting results. In DPPH radical assay both extracts exhibited good quenching abilities but alkaline hydrolyzable fraction was much more efficient with 82.2% of neutralized DPPH radicals. On the other hand, extract that contained extractable phenolic fraction demonstrated excellent possibilities to neutralize 81.4% of artificial ABTS radical cation model while the extract that contained alkaline hydrolyzable phenolic fraction possessed significantly reduced ABTS radical cation scavenging activity—about 15%. This opposite trend can be related to different composition of two phenolic fractions as well as to possible differences in the composition of accompanying compounds. Namely, alkaline hydrolyzable fraction of phenolics contained almost exclusively ferulic acid which was not detected among extractable phenolic compounds. It was documented that phenolic acids and particularly this acid have good ability to quench DPPH radical, even better compared to some standard compounds such as BHT, α-tocopherol or ascorbic acid [59,60]. During strong alkaline and acidic conditions applied for releasing alkaline hydrolyzable phenolics it was possible that hydrolysis of some proteins occurred leading to the production of bioactive peptides which are also capable to neutralize free radicals [61,62]. Decreased DPPH radical scavenging activity for extractable phenolic fraction can be explained with the fact that, in some cases, DPPH radical gives reversible reaction with phenolic compounds which can lead to lower results than expected [63]. Furthermore, although both are radicals it is well known that ABTS radical cation is more hydrophilic while DPPH radical is more lipophilic compound [55]. As support for this statement, lipid fraction obtained from pollen exhibited some moderate activity against DPPH radical (22.3% of inhibition) but negligible ability to quench ABTS radical cations (4.8% of inhibition). Results for ABTS radical cation assay for extractable phenolic fraction are slightly lower but comparable or in line with results presented in literature for different pollen samples: 95.5% for monofloral sunflower bee-collected pollen from Serbia [7], 86.9% and 80.4% for rapeseed and coconut Indian monofloral bee-collected pollen, respectively [8]. In addition, as positive control, ascorbic acid solution with the same concentration as pollen extracts (0.1 g/mL) was prepared and used in these quenching assays. The obtained results were as follows: 93.9% of inhibition for DPPH radicals as well as 99.0% of inhibition for ABTS radical cation. Based on this it can be concluded that extractable, i.e., alkaline hydrolyzable phenolic fractions can be marked as a good source of compounds able to quench ABTS and DPPH radicals, respectively.

The fifth applied antioxidant assay in the current study, FCC, revealed that pollen did not possess a significant ability to form complexes with Fe^2+^ ions—13.3% for extractable and 14.2% for alkaline hydrolyzable fraction. The obtained results were not significantly different (*p* < 0.05) and were significantly lower compared to the results for Indian bee-collected pollen samples (44.0–84.6%) [8]. However, it can be related to different botanical origin of pollen, different content of proteins, which are recognized as the most important chelating agents, as well as possibilities that some other metal ions present in artichoke pollen occupy the bounding places in proteins during competition with Fe^2+^ ions. Interestingly, in this assay non-significant difference was observed between extractable and alkaline hydrolyzable fractions in contrast with the other assays. The possible explanation can be that, during the digestion of the sample to obtain alkaline hydrolyzable fraction, strong alkaline and acidic conditions provoked release of some peptides with chelating abilities [64] which compensated for reduced phenolic and/or protein content in this fraction.

Summarizing the given results, bee-collected artichoke pollen exhibited good antioxidant properties by studying three obtained fractions (lipid, extractable phenolic and alkaline hydrolysable phenolic) using five different in vitro screening antioxidant assays. For the most of applied assays extractable phenolic fraction was the best source of antioxidants. It should be pointed out that these in vitro assays are time-demanding and sometimes expensive since at least three of them must be performed to obtain reliable results [55]. Nevertheless, further *in vivo* experiments on bee-collected artichoke pollen are more than desirable in order to connect the obtained data with possible health benefits for consumers.

## Figures and Tables

**Table 1:** Phytochemical composition (mg/kg dry weight) in bee-collected artichoke pollen.

Compound	Extractable Fraction	Alkaline Hydrolyzable Fraction
TPC * (mg/kg GAE ^1^ DW)	5314.2 ± 60.9 ^a,A^	497.9 ± 3.8 ^b,A^
TFC * (mg/kg QE ^1^ DW)	812.0 ± 82.9 ^a,B^	957.0 ± 145.0 ^a,B^
TCD * (mg/kg CGAE ^1^ DW)	1064.7 ± 80.3 ^C^	n.d. ^1^
TC (mg/kg DW)	5.00 ± 0.20	

* TPC—Total phenolic content; TFC—total flavonoid content; TCD—total dihydroxycinnamic acid derivative content; TC—total carotenoid content. ^1^ GAE—gallic acid equivalents; QE—quercetin equivalents; CGAE—chlorogenic acid equivalents; DW—dry weight; n.d.—not detected. Results are presented as mean ± SD; (n = 3). Values carrying different lowercase letters in the same row are significantly different (*p* < 0.05) according to *t*-test. Values carrying different uppercase letters in the same column are significantly different (*p* < 0.05) according to *t*-test.

**Table 2:** Phenolic content (mg/kg dry weight) in bee-collected artichoke pollen.

Compound	Extractable Fraction	Alkaline Hydrolyzable Fraction
Phenolic acids		
Caffeic acid	0.416 ± 0.019	n.d. ^1^
Ferulic acid	n.d.	30.393 ± 1.586
Σ	0.416 (0.66) *	30.393 (83.28)
Flavonols		
Quercetin 3-*O*-glucoside (Isoquercetin)	1.836 ± 0.064	n.d.
Rutin	3.662 ± 0.158 ^a^	0.655 ± 0.019 ^b^
Isorhamnetin 3-*O*-glucoside	49.171 ± 1.679 ^a^	4.613 ± 0.128 ^b^
Kaempferol	1.527 ± 0.058	n.d.
Astragalin (Kaempferol-3-*O*-glucoside)	2.508 ± 0.032 ^a^	0.189 ± 0.005 ^b^
Σ	58.704 (92.41)	5.457 (14.95)
Other detected phenolics		
Apigenin	2.633 ± 0.060 ^a^	0.373 ± 0.014 ^b^
Apigetrin (Apigenin-7-*O*-glucoside)	1.332 ± 0.084 ^a^	0.274 ± 0.019 ^b^
Aesculetin	0.438 ± 0.025	n.d.
Σ	4.403 (6.93)	0.647 (1.77)
Total sum	63.523	36.497

^1^ n.d.—not detected. * Values in parentheses represent a relative amount of phenolic class in the extractable and alkaline hydrolyzable fraction of bee-collected artichoke pollen. Results are presented as mean ± SD; (n = 3). Values carrying different letters in the same row are significantly different (*p* < 0.05) according to *t*-test.

**Table 3:** Fatty acid content (% of total fatty acids expressed in dry weight) in bee-collected artichoke pollen with nutritional parameters.

Compound/Parameter	The Content
C12:0	10.50 ± 0.60 ^a^
C14:0	1.20 ± 0.08 ^b^
C14:1	8.95 ± 0.53 ^c^
C15:0	11.46 ± 0.45 ^a^
C16:0	0.50 ± 0.03 ^d^
C16:1	2.57 ± 0.18 ^e^
C17:1	1.30 ± 0.09 ^b^
C18:1n9c	24.90 ± 1.45 ^f^
C18:2n6c	6.60 ± 0.23 ^g^
C20:2	3.60 ± 0.02 ^h^
C20:5n3	28.40 ± 1.1 ^i^
nutritional parameters	
UFA ^1^	76.32
MUFA	37.72
PUFA	38.60
SFA	23.66
ω-6	10.20
ω-3	28.40
UFA/SFA ratio	3.23
ω-6/ω-3 ratio	0.36

C12:0—lauric acid; C14:0—myristic acid; C14:1—miristoleic acid; C15:0—pentadecanoic acid; C16:0—palmitic acid; C16:1—palmitoleic acid; C17:1—*cis*-10-heptadecanoic acid; C18:1n9c—oleic acid; C18:2n6c—linoleic acid; C20:2—*cis*-11,14-eicosadienoic acid; C20:5n3—*cis*-5,8,11,14,17-eicosapentaenoic acid (EPA). Results are presented as mean ± SD; (n = 3). Values carrying different letters in the column are significantly different (*p* < 0.05) according to *t*-test. ^1^ UFA—unsaturated fatty acids; MUFA—monounsaturated fatty acids; PUFA—polyunsaturated fatty acids; SFA—saturated fatty acids.

**Table 4:** Antioxidant properties of phenolic and lipid fractions of bee-collected artichoke pollen.

Assay	Extractable Fraction	Alkaline Hydrolyzable Fraction	Lipid Fraction
* TAC (mg/kg AAE ^1^ DW)	21,912.4 ± 1118.1 ^a^	813.0 ± 20.8 ^c^	7830.9 ± 425.6 ^b^
* FRP (mg/kg AAE ^1^ DW)	468.6 ± 12.3 ^a^	115.1 ± 1.6 ^b^	n.d.
* ABTS^∙+^ (% of inhibition)	81.41 ± 0.88 ^a^	14.74 ± 0.11 ^b^	4.80 ± 2.18 ^c^
* DPPH∙ (% of inhibition)	38.30 ± 0.77 ^b^	82.23 ± 0.33 ^a^	22.32 ± 0.39 ^c^
* FCC (% of chelating ability)	13.31 ± 0.55 ^a^	14.25 ± 0.72 ^a^	n.a.

* TAC—total antioxidant capacity; FRP—ferric reducing capacity; ABTS**^∙+^**-2,2′-azino-bis-(3-ethylbenzenthiazoline-6-sulfonic acid) radical cation; DPPH—2,2-diphenyl-1-picrylhydrazyl radical; FCC—ferrous chelating capacity. ^1^ AAE—ascorbic acid equivalents; DW—dry weight; n.d.—not detected; n.a.—not applicable. Results are presented as mean ± SD; (n = 3). Values carrying different letters in the same row are significantly different (*p* < 0.05) according to *t*-test.

## Data Availability

No new data were created or analyzed in this study. Data sharing is not applicable to this article.

## Supplementary Materials

The following are available online at https://www.mdpi.com/article/10.3390/antiox10071091/s1, Figure S1: UV chromatograms at 280 nm with the main marked peaks of: (A) extractable phenolic fraction and (B) alkaline hydrolyzabile phenolic fraction, Table S1: MS data used for phenolic identification and quantification.
