# Supplementary material for: Phytochemical Profile and Antioxidant Properties of Bee-Collected Artichoke (Cynara scolymus) Pollen
Source: Antioxidants (Basel). 2021 Jul 7;10(7):1091. doi: 10.3390/antiox10071091 (PMC8301145; doi:10.3390/antiox10071091)
Supplement: Supplementary file 1 [file antioxidants-10-01091-s001.zip › antioxidants-1261081-supplementary.pdf]

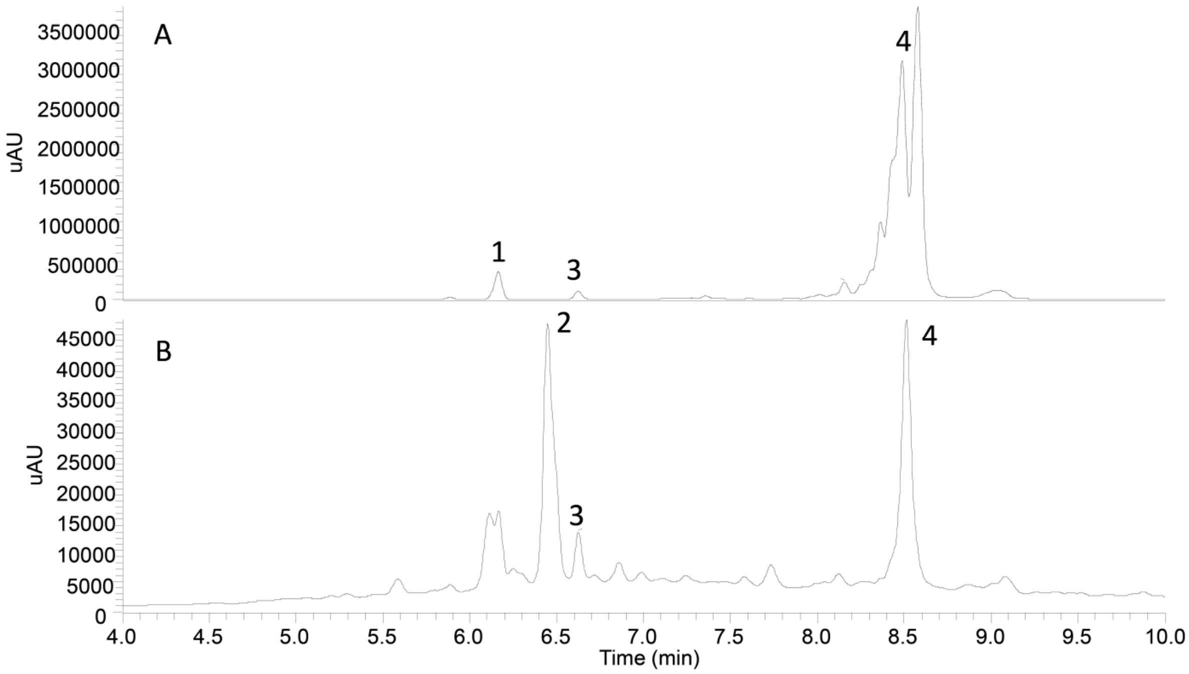

Figure S1. UV chromatograms at 280 nm with the main marked peaks of: (**A**) extractable phenolic fraction and (**B**) alkaline hydrolyzabile phenolic fraction; Legend: 1 – Quercetin-3-*O*-glucoside, 2 – Kaempferol-3-*O*-glucoside; 3 – Isorhamnetin-3-*O*-glucoside, and 4 – Apigenin.

Table S1. MS data used for phenolic identification and quantification.

Compounds	Retention Time, *min*	Parent Ion, $[M–H]^-$, *m/z*	Product Ions, $[M–H]^-$, *m/z* (Collision Energy, *eV*)
**Caffeic acid**	5.33	179.004	134.00 (13); 135.00 (16)
**Ferulic acid**	6.54	193.057	134.00 (18); 178.00 (15)
**Isoquercetin (Quercetin-3-*O*-glucoside)**	6.19	463.002	271.01 (44); 300.02 (29)
**Rutin**	6.03	609.197	299.98 (42); 301.20 (32)
**Isorhamnetin-3-*O*-glucoside**	6.63	477.009	314.02 (28); 314.98 (36)
**Kaempferol**	8.80	285.074	211.00 (32); 227.00 (32)
**Astragalin (Kaempferol-3-*O*-glucoside)**	6.55	447.088	257.03 (40); 285.03 (30)
**Apigenin**	8.53	269.032	117.24 (43); 149.00 (24)
**Apigetrin (Apigenin-7-*O*-glucoside)**	6.68	431.004	239.11 (53); 268.03 (36)
**Aesculetin**	5.30	176.911	133.08 (21); 153.00 (20)
